# Dynamic inversion of planar-chiral response of terahertz metasurface based on critical transition of checkerboard structures

**DOI:** 10.1515/nanoph-2021-0671

**Published:** 2022-01-11

**Authors:** Yoshiro Urade, Kai Fukawa, Fumiaki Miyamaru, Kunio Okimura, Toshihiro Nakanishi, Yosuke Nakata

**Affiliations:** Department of Electronic Science and Engineering, Kyoto University, Kyoto 615-8510, Japan; Department of Physics, Shinshu University, Nagano 390-8621, Japan; School of Engineering, Tokai University, Hiratsuka, Kanagawa 259-1292, Japan; Graduate School of Engineering Science, Osaka University, Osaka 560-8531, Japan; Center for Quantum Information and Quantum Biology, Osaka University, Osaka 560-8531, Japan

**Keywords:** asymmetric transmission, circular conversion dichroism, metasurface, planar chirality, terahertz photonics, vanadium dioxide

## Abstract

Dynamic inversion of the planar-chiral responses of a metasurface is experimentally demonstrated in the terahertz regime. To realize this inversion, the critical transition of the checkerboard-like metallic structures is used. Resonant structures with planar chirality and their complementary enantiomeric patterns are embedded in the checkerboard. Using vanadium dioxide as a variable resistance, the metasurface is implemented in the terahertz regime. The responses of the metasurface to circularly polarized waves are then characterized by terahertz time-domain spectroscopy. Further, the sign of the circular conversion dichroism, which is closely related to the handedness of the planar chirality of the metasurface, is observed to be inverted at 0.64 THz by varying the temperature. Such invertible planar-chiral responses can be applied practically to the handedness-invertible chiral mirrors.

## 1 Introduction

Artificial materials composed of subwavelength structures, such as metamaterials and metasurfaces, have interesting electromagnetic properties, such as bianisotropy [1], negative refractive index [2, 3], and hyperbolic dispersion [4]. In addition, “asymmetric transmission” has also been discovered in research on artificial materials [5]. The total transmission intensities of circularly polarized waves through anisotropic and lossy metasurfaces depend on the direction of incidence. This seemingly nonreciprocal phenomenon does not contradict the Lorentz reciprocity theorem [6, 7] and has also been observed in linearly polarized waves [8]. Asymmetric transmissions of circularly polarized waves are equivalent to the circular conversion dichroism (CCD), which represents the difference in the cross-polarized transmission efficiency of circularly polarized waves, and are associated with the “planar chirality” property of metasurfaces [5, 9], [10], [11]. A two-dimensional structure is considered to be planar chiral if it has no line of mirror symmetry. Due to this broken symmetry, the distribution of the induced current on planar-chiral metasurfaces for incidence of left circularly polarized waves is not obtained by mirror reflection of that for incidence of right circularly polarized waves. Consequently, the helicity-changing transmission efficiency can be different for each handedness. The sign of the asymmetric transmission or CCD corresponds to the handedness of planar chirality. By combining planar-chiral metasurfaces with normal mirrors, novel chiral mirrors that can absorb only one handedness of the circular polarization and reflect the other have been realized [12–14]. Holography based on chiral mirrors has also been demonstrated in the terahertz regime [15].

Recent achievements in reconfigurable metasurfaces have enabled dynamic tuning of planar-chiral responses, such as asymmetric transmissions, using graphene [16–19], vanadium dioxide (VO_2_) [20, 21], and flexible substrates [22]. Reconfigurable chiral mirrors, which can switch between the two handedness states, have also been realized in the terahertz regime [14]. While these reported works have achieved novel tunability and reconfigurability through carefully designed structures and material parameters, inversion of the planar-chiral responses at the *identical* frequency has not been achieved yet. For example, handedness inversion has been realized for chiral mirrors [14], but the frequencies at which the planar-chiral responses manifest are different for the two handedness states. For applications such as switchable circular polarizers, it is desirable that inversion occur exactly at the same band for the operation with the narrow-band light sources. Hence, a new design methodology to realize exact inversion of planar-chiral responses needs to be established. However, exact inversion of planar-chiral responses requires large structural deformations, where structures with one handedness must be deactivated and their enantiomeric structures must be activated. Furthermore, modification by active materials such as VO_2_ is additive such that the metallic structures cannot be eliminated, and the metallic regions can only be extended by insulator-to-metal transition of VO_2_. Thus, inversion of planar-chiral responses at the identical frequencies has been challenging thus far.

To this end, the focus here is on the critical behaviors of the checkerboard-like metallic structures [23–28]. Their electromagnetic responses change drastically based on the connectivities of the metallic parts. According to Babinet’s principle [26], the responses in the connected and disconnected phases are closely related. Based on this characteristic, the checkerboard-like metasurfaces were integrated with VO_2_, which undergoes metal–insulator transition at around 340 K [29], to realize capacitive–inductive switchable filters [30], switchable linear polarizers [31], and reconfigurable quarter-wave plates [32, 33]. Moreover, it was theoretically shown that the asymmetric transmission of circularly polarized waves can be inverted over the entire spectrum by imposing special geometric arrangements on the checkerboard-like metasurfaces [34]. The primary advantage of the proposed design principle is that metasurfaces can be easily designed to show the inversion at the identical frequencies without fine tuning the geometrical parameters, which is beneficial for further performance optimization. In the present study, the inversion of planar-chiral responses (CCD) is experimentally demonstrated at the identical frequency based on checkerboard-like metasurfaces.

The remainder of this paper is structured as follows. Section 2 briefly reviews the principle of dynamic inversion of the planar-chiral responses of checkerboard-like metasurfaces with planar chirality. Section 3 presents the experiments performed at terahertz frequencies along with a discussion of the results. Section 4 summarizes the results obtained and the future outlook.

## 2 Principle

**Figure 1:**
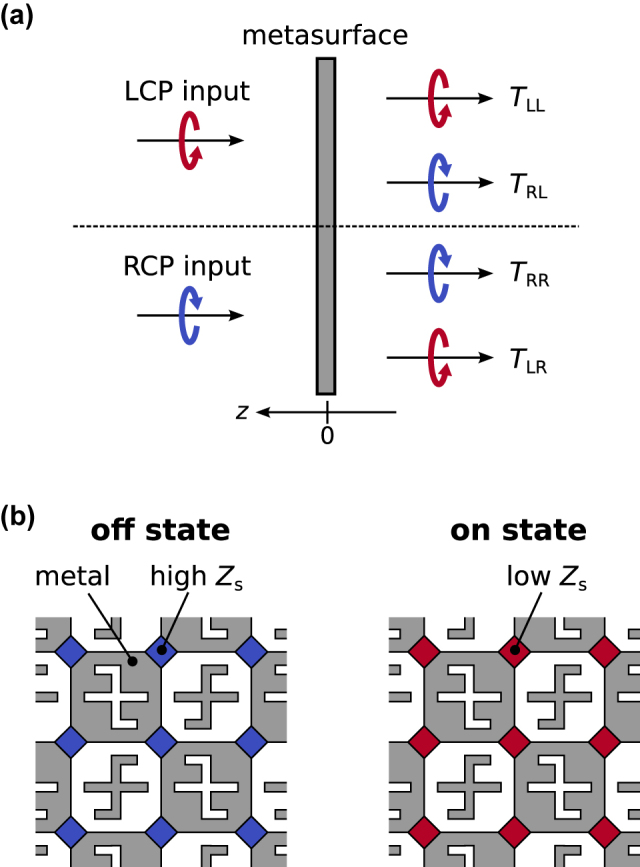
(a) Metasurface is placed on the *z* = 0 plane in vacuum. The LCP and RCP plane waves are incident on the metasurface from *z* > 0, and *T*_*ij*_ denotes the power transmission through the metasurface (*i*: output mode, *j*: input mode). (b) Schematic of a checkerboard-like metasurface with planar chirality in the “off” (*Z*_s_ > *Z*_0_/2) and “on” (*Z*_s_ < *Z*_0_/2) states.

This section briefly reviews the principle of dynamic inversion of the planar-chiral responses of checkerboard-like metasurfaces, which is also discussed in our previous theoretical work [34]. First, the idea of asymmetric transmission for circularly polarized waves is introduced by considering a metasurface placed on the *z* = 0 plane in vacuum, as shown in Figure 1a. The metasurface is irradiated by left circularly polarized (LCP) or right circularly polarized (RCP) plane waves from the *z* > 0 side along the normal direction.1In this work, we use the polarization bases $e_{+}=\left(e_{x}+je_{y}\right)/\sqrt{2}$ and $e_{-}=\left(e_{x}-je_{y}\right)/\sqrt{2}$ for the LCP and RCP waves incident from *z* > 0, respectively. *e*_*x*_ (*e*_*y*_) and j denote the unit vector in the *x* (*y*) direction and imaginary unit, respectively. Note that the polarization basis vectors are interchanged if the LCP and RCP waves are incident from *z* < 0. It is also noted that the harmonic time dependence of exp(j*ωt*) is assumed herein. Scattering into other modes with different wave vectors (except for specular reflection) is assumed to be negligible. This assumption is valid if the periodicity of the metasurface is smaller than the wavelength of the incident wave. Thus, there are only two modes in the transmission, namely the co-polarized and cross-polarized modes. *T*_*ij*_ in Figure 1a denotes the power transmission to these modes through the metasurface (*i*: output mode, *j*: input mode). The power transmission in the case of incidence from the *z* < 0 side is denoted similarly.

Asymmetric transmission Δ*T* is defined by the directional difference of the total transmission for incidence of the LCP waves [5] as follows:

(1) $$\Delta T=\left(T_{\text{LL}}^{>}+T_{\text{RL}}^{>}\right)-\left(T_{\text{LL}}^{<}+T_{\text{RL}}^{<}\right),$$

where the superscript > ( < ) denotes incidence from the *z* > 0 (*z* < 0) side. From the Lorentz reciprocity theorem [6, 7], $T_{\text{LL}}^{>}=T_{\text{LL}}^{<}$ and $T_{\text{RL}}^{<}=T_{\text{LR}}^{>}$ ; thus, Eq. (1) reduces to

(2) $$\Delta T=T_{\text{RL}}^{>}-T_{\text{LR}}^{>}.$$

Note that Δ*T* is expressed using only the transmission waves incident from the *z* > 0 side. In other words, Δ*T* is equivalent to the CCD; therefore, the superscript > is omitted for simplicity from this point onwards. The sign of Δ*T* corresponds to the handedness of the planar chirality of the metasurface, i.e., its enantiomer in terms of the planar chirality results in a Δ*T* with the opposite sign. Note that circular dichroism, the difference in co-polarized components $T_{\text{LL}}^{>}-T_{\text{RR}}^{>}$ , vanishes due to mirror symmetry in the *z* direction and the Lorentz reciprocity.

Next, a checkerboard-like metasurface with planar chirality is introduced as shown in Figure 1b. It is assumed that the checkerboard-like metasurface is placed on the *z* = 0 plane in vacuum, as depicted in Figure 1a, and consists of checkerboard-like metallic patterns wherein the anisotropic gammadion-like resonators [35, 36] and their complementary holes with opposite handedness are embedded. This special arrangement of the embedded structures is crucial to the inversion of the planar-chiral responses. Herein, the metallic parts are assumed to be approximated as perfect electric conductors. The metallic patches are connected to each other via variable-impedance sheets with impedance *Z*_s_. By controlling *Z*_s_, the sign of Δ*T* of the present metasurface can be inverted.

The metasurface is said to be in its “off” (“on”) state if *Z*_s_ > *Z*_0_/2 (*Z*_s_ < *Z*_0_/2), where *Z*_0_ ≈ 377 Ω is the impedance of vacuum. From Babinet’s principle [26] and the special geometric arrangement of the metasurface, there exists a one-to-one correspondence between the two states [34] as

(3) $$\Delta T_{\text{on}}=-\Delta T_{\text{off}},$$

if $Z_{s,on}={\left(Z_{0}/2\right)}^{2}/Z_{s,o\mathrm{ff}}$ . In other words, by controlling *Z*_s_ across *Z*_0_/2, the sign of Δ*T* over the entire spectrum can be flipped. Note that this inversion can be regarded as inversion of the planar chirality of the metasurface by defining the planar chirality with the sign of Δ*T*.

It is noted that the above discussion is valid only when Babinet’s principle is applicable [37], i.e., the metals in the metasurfaces can be treated as perfect electric conductors with negligible thicknesses and the dielectric environments above and below the metasurfaces are mirror symmetric [26]. In general experiments, the latter condition cannot be satisfied in many cases owing to the dielectric substrates that support metasurfaces. As shown by the numerical simulations in [34]; however, the sign inversion of Δ*T* in the checkerboard-like metasurface occurs even with the dielectric substrate. Thus, a metasurface is designed herein on the basis of the present theory. It is noted that the Lorentz reciprocity theorem is valid even in the presence of dielectric materials; therefore, the definition of Δ*T* in Eq. (2) does not change for a metasurface on a dielectric substrate.

It should be noted that a planar-chiral structure located on a dielectric substrate has three-dimensional chirality in terms of symmetry, since the substrate breaks mirror symmetry in the *z* direction. However, it has been experimentally confirmed that circular dichroism of such metasurfaces is small and the planar-chiral structures on the substrates determine their responses [5, 11].

## 3 Experiments at terahertz frequencies

The dynamic inversion of the CCD in the checkerboard-like metasurface is experimentally demonstrated at terahertz frequencies. Metals such as aluminum (Al) are good electric conductors in this frequency regime and can thus be approximated as perfect electric conductors, as noted in Section 2. Further, the metal–insulator transition of VO_2_ can be used to realize the variable resistance for dynamic inversion. In the following sections, the details of the design of the metasurface, numerical simulations, and terahertz spectroscopy are described.

### 3.1 Design and fabrication

**Figure 2:**
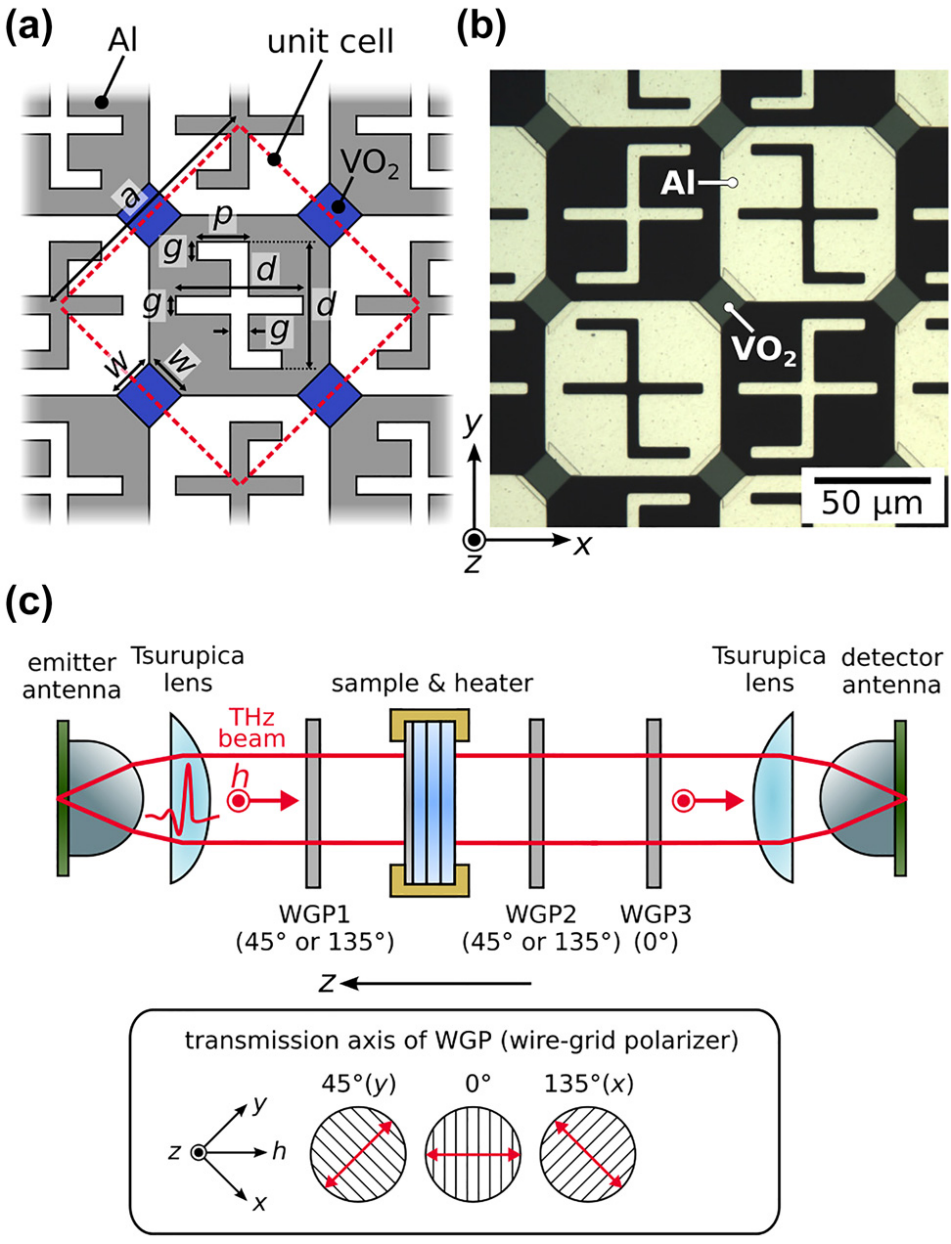
(a) Schematic of the designed metasurface. The red dashed square indicates the unit cell of the metasurface with dimensions as follows: *a* = 106 μm, *d* = 60 μm, *g* = 5 μm, *p* = 27 μm, and *w* = 15 μm. (b) Photomicrograph of the fabricated metasurface, with patterns repeated over a 14-mm square area on the substrate. (c) Schematic of the experimental setup for terahertz time-domain spectroscopy. The emitted terahertz waves are linearly polarized in the *h* direction, and the inset shows the transmission axis of the WGPs in the setup.

The metasurface design is based on the principle shown in Section 2. The schematic and dimensions of the metasurface are shown in Figure 2a. The dimensions are as follows: *a* = 106 μm, *d* = 60 μm, *g* = 5 μm, *p* = 27 μm, and *w* = 15 μm. These parameters are determined via finite element simulations of the metasurface (see Subsection 3.2 for details) so that the resonance of the anisotropic gammadion-like structures occurs in the measurable frequency range around 0.5 THz.

The designed metasurface structure is fabricated on a *c*-plane sapphire substrate of thickness 1 mm. The procedure for fabrication is the same as that used in [30]. A VO_2_ film of thickness approximately 150 nm is grown on the substrate by reactive magnetron sputtering of a vanadium target. The VO_2_ film is patterned by photolithography and wet etching. Then, the structure made of Al (thickness: 400 nm) is formed by photolithography, electron-beam evaporation, and subsequent lift-off. The photomicrograph of the fabricated metasurface is shown in Figure 2b. The Al patterns and VO_2_ patches have overlaps to ensure adequate electric connections. Such patterns are repeated over a 14-mm square area on the substrate.

### 3.2 Numerical simulation

Numerical simulations were performed to design and evaluate the metasurface by the finite element method (COMSOL Multiphysics). The metasurface was modeled by the transition boundary condition in COMSOL Multiphysics: conductivity *σ*_Al_ = 22 S/μm [38] and thickness *t*_Al_ = 400 nm for the Al part, and $\sigma_{{VO}_{2}}=1/\left(Z_{s}t_{{VO}_{2}}\right)$ and $t_{{VO}_{2}}=200\;nm$ for the VO_2_ part. The metasurface structure was formed at the interface between vacuum and an anisotropic dielectric material with refractive index *n* = 3.1 in directions parallel to the metasurface and *n*_*z*_ = 3.4 for the orthogonal direction, which models the *c*-plane sapphire substrate [39]. Periodic boundary conditions were imposed on the unit cell of the metasurface to simulate the responses of the entire metasurface. Power transmission for circularly polarized waves is then calculated for the input and output ports located in vacuum and the dielectric material. The calculated transmissions are normalized by the power transmission of the vacuum–sapphire interface without the metasurface, *T*_sapp_ = 4*n*/(1 + *n*)^2^, for comparisons with measurements.

### 3.3 Characterization by terahertz time-domain spectroscopy

The responses of the metasurface are characterized by conventional terahertz time-domain spectroscopy. To obtain the transmission spectra for the circularly polarized waves, the Jones matrix [40, 41] is first measured for the linearly polarized waves:

(4) $$M_{\text{lin}}=\left(\begin{array}{cc} t_{xx} & t_{xy}\\ t_{yx} & t_{yy} \end{array}\right),$$

where *t*_*ij*_ represent the complex coefficients of transmission from the *j* polarization to *i* polarization. Then, the base is changed from linear to circular [11] as follows:

(5) $$\begin{array}{cc} M_{\text{circ}} & =\left(\begin{array}{cc} t_{\text{LL}} & t_{\text{LR}}\\ t_{\text{RL}} & t_{\text{RR}} \end{array}\right)\\ & =\Lambda^{-1}M_{\text{lin}}\Lambda , \end{array}$$

where Λ is the transformation matrix for basis change given by

(6) $$\Lambda =\frac{1}{\sqrt{2}}\left(\begin{array}{cc} 1 & 1\\ j & -j \end{array}\right).$$

This transformation suggests that the CCD and equivalently Δ*T* of the metasurface can be characterized using a linearly polarized source of terahertz waves.

The schematic of the experimental setup is shown in Figure 2c. Dipole-type photoconductive antennas excited by femtosecond laser pulses are used for the emitter and detector of the terahertz pulses. The emitted terahertz waves are linearly polarized in the *h* direction, as shown in Figure 2c. The collimated terahertz pulses pass through an aperture (not shown in the schematic), three wire-grid polarizers (WGPs), and the metasurface, before being detected at the other photoconductive antenna. The angle of WGP1 determines the incident linear polarization (*x* or *y*), and WGP2 selects the detected polarization. WGP3 projects both polarization states onto the same polarization state for detection by the photoconductive antenna. The definitions of the angles of the WGPs are shown in the inset of Figure 2c, where 0° corresponds to the transmission axis along the *h* direction.

The metasurface is placed in a sample holder with an electric heater, whose temperature *T*_hold_ is monitored using a thermocouple attached to the holder. The temperature can be stabilized by feedback to the current in the heater. The temperature variation is within ±1 K. In general, *T*_hold_ and the actual temperature of the sample are different, so the set temperature for *T*_hold_ is compensated in the feedback control to achieve the target temperature of the sample. Before the terahertz measurements, the temperature of the sapphire substrate, *T*_subst_, is measured using another thermocouple directly attached to the substrate. Based on the difference between *T*_hold_ and *T*_subst_, the set temperature for *T*_hold_ is compensated in terahertz time-domain spectroscopy. The temperature values shown below are corrected by this result.

Two more *c*-plane sapphire substrates were added beneath the metasurface substrate in the sample holder. By thickening the substrate, multiple reflection pulses caused by the air–substrate interface can be separated in the time domain. To use as reference in terahertz time-domain spectroscopy, stacked *c*-plane sapphire substrates with the same thickness as the sample were also placed in the sample holder.

The procedure for the measurements is as follows:

1. WGP1 is set at 45°.
2. Reference signal is measured after WGP2 is set at the same angle as WGP1.
3. Measure the metasurface signal.
4. Measure the metasurface signal after setting WGP2 at an angle orthogonal to WGP1.
5. Repeat 2–4 with WGP1 set at 135°.

Each normalized transmission coefficient is calculated by ${\hat{t}}_{ij}={\tilde{E}}_{ij}^{\left(meta\right)}/{\tilde{E}}_{j}^{\left(ref\right)}$ , where ${\tilde{E}}_{ij}^{\left(meta\right)}$ and ${\tilde{E}}_{j}^{\left(ref\right)}$ represent the Fourier transforms of the time-domain signals measured by the detector antenna (*i*: detected polarization, *j*: incident polarization) for the metasurface and reference, respectively. These measurements are performed repeatedly while changing the temperature of the metasurface. Stabilizing the temperature takes several minutes before starting the measurements. For each stabilized temperature, the procedure is repeated 10 times, and the obtained transmission coefficients are averaged. Then, the circular transmission coefficients are obtained by the transformation shown in Eq. (5).

### 3.4 Experimental results

**Figure 3:**
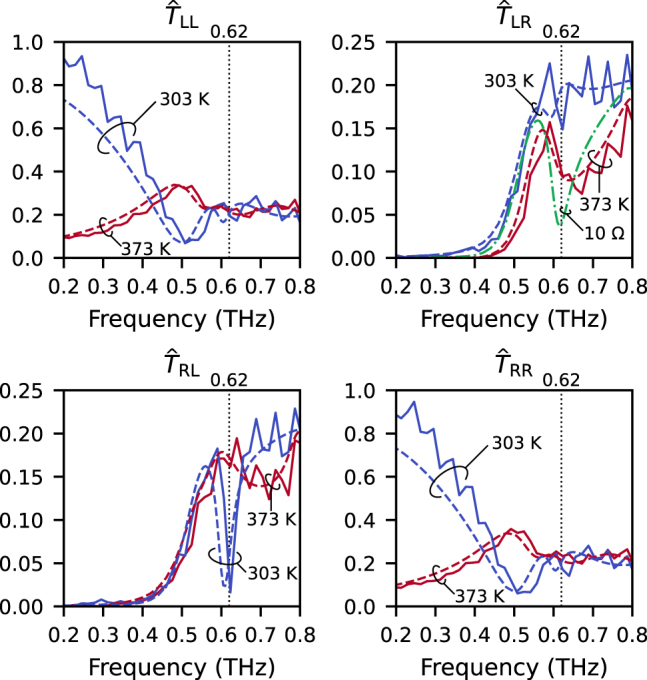
Normalized power transmission spectra of the metasurface for circularly polarized waves at 303 and 373 K. The solid and dashed curves show the experimental and numerically simulated results, respectively. The sheet impedances *Z*_s_ used to model the VO_2_ film in the simulation are 1000 Ω for 303 K and 30 Ω for 373 K, respectively. The green dashed-dotted line in ${\hat{T}}_{\text{LR}}$ shows the simulated spectrum for *Z*_s_ = 10 Ω.

Figure 3 shows the normalized power transmission spectra ${\hat{T}}_{ij}≔{\left|{\hat{t}}_{ij}\right|}^{2}$ of the metasurface at 303 and 373 K. It is confirmed that ${\hat{T}}_{\text{LL}}$ and ${\hat{T}}_{\text{RR}}$ spectra change drastically according to temperature; this is because insulator–metal transition of VO_2_ occurs for the increasing temperatures, and the checkerboard-like metasurface consequently undergoes phase transition [30]. The dashed curves in Figure 3 show the results of the numerical simulations. The sheet impedances *Z*_s_ used to model the VO_2_ film in the simulation are 1000 Ω for 303 K and 30 Ω for 373 K, and these show good agreement with the experimental results. It is noted that the lowest diffraction frequency of the present metasurface is *f*_d_ = *c*_0_/(*n*_*z*_ *a*) = 0.83 THz (*c*_0_: velocity of light in vacuum), and only the zeroth-order diffraction contributes to the transmission in this observation range.

A sharp dip is noted around 0.62 THz in the ${\hat{T}}_{\text{RL}}$ spectrum at 303 K, and a similar dip structure that is broader and shallower is also noted in the ${\hat{T}}_{\text{LR}}$ spectrum at 373 K. For simulation with *Z*_s_ = 10 Ω (shown by the green dashed-dotted line in ${\hat{T}}_{\text{LR}}$ ), a sharper dip is confirmed around 0.62 THz in the ${\hat{T}}_{\text{LR}}$ spectrum. These dip structures originate from the resonances of the gammadion-like structures and contribute to the large CCD. Such microscopic origins of the spectra have been thoroughly discussed in the previous theoretical investigation [34].

It is noted that ${\hat{T}}_{\text{LL}}$ and ${\hat{T}}_{\text{RR}}$ show almost similar spectra regardless of the temperature, indicating that the optical activity (circular dichroism, quantified by ${\hat{T}}_{\text{LL}}-{\hat{T}}_{\text{RR}}$ ), which is associated with the three-dimensional chirality, of the sample is small, although the substrate endows three-dimensional chirality to the sample [41].

**Figure 4:**
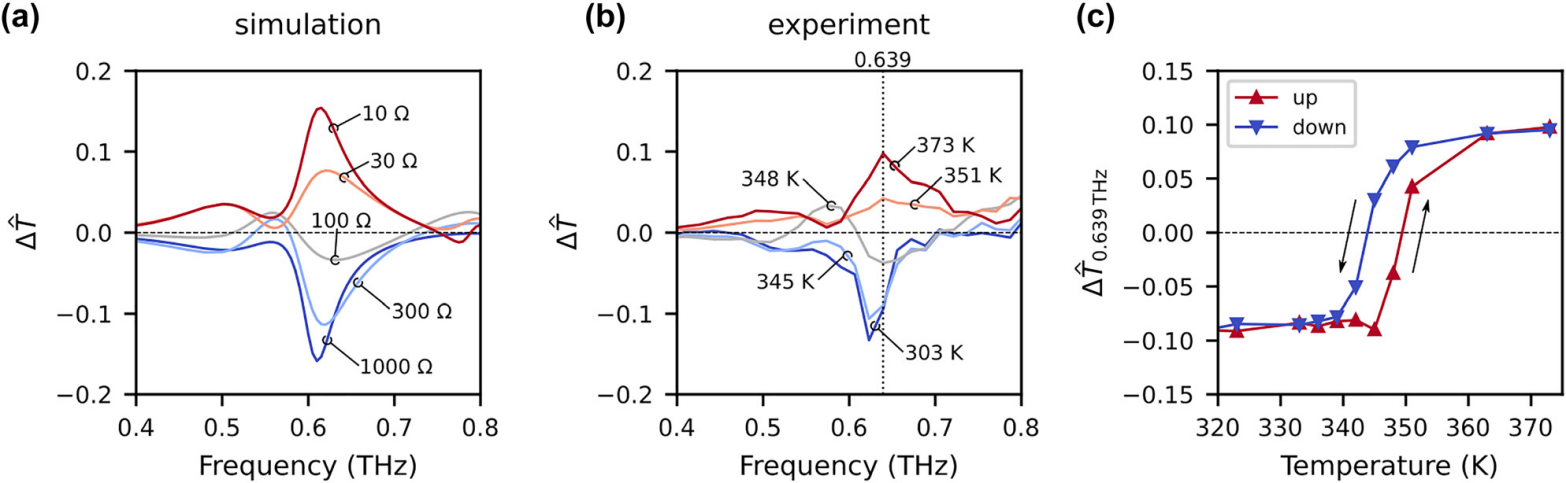
(a) Simulated $\Delta \hat{T}$ spectra of the metasurface for a set of sheet impedances *Z*_s_. (b) Measured $\Delta \hat{T}$ spectra of the metasurface for a set of temperatures. These data are taken when heating the sample from ambient temperature to 373 K. (c) Temperature dependence of $\Delta \hat{T}$ at 0.639 THz. The data for both heating and cooling of the sample are plotted.

Figure 4a and b show the $\Delta \hat{T}$ spectra for the numerical simulations and experiments, respectively. Here, $\Delta \hat{T}≔\Delta T/T_{\text{sapp}}$ . For the simulations, a set of sheet impedances *Z*_s_ were used to mimic the resistance changes of the VO_2_ film. For the experiments, the data were obtained from the heating condition of the sample from ambient temperature ( ≈295K ) to 373 K. In both cases, it is confirmed that the sign of $\Delta \hat{T}$ , namely CCD, is flipped by changing the parameters. It is also confirmed that the metasurface switches from the off to on states; this sign inversion can be interpreted as inversion of the planar chirality of the metasurface.

The temperature dependence of $\Delta \hat{T}$ at 0.639 THz is shown in Figure 4c, where data for both heating and cooling of the sample are plotted. In the case of heating, the metasurface is gradually heated from ambient temperature to 373 K. For cooling, the metasurface is heated to 373 K and then gradually cooled to ambient temperature. Clear thermal hysteresis, which is typically seen for the resistivity of VO_2_ [29], is observed. In other words, the present metasurface has a memory effect [42], which is inherited from VO_2_, in its planar-chiral response.

## 4 Conclusions

In conclusion, the dynamic inversion of the CCD of the planar-chiral metasurface is experimentally demonstrated in the terahertz regime. Owing to the checkerboard-like geometry and according to Babinet’s principle, the metasurface can be easily designed so that inversion occurs at the identical frequency; this is important for further optimization of the planar-chiral responses of invertible metasurfaces. The metasurface in this work was realized using metal–insulator transition of VO_2_. It is shown that the CCD $\Delta \hat{T}$ , whose sign characterizes the handedness of the planar chirality of the structure, can be continuously controlled within a range of ±0.1 at 0.64 THz along with the memory effect. The tunable range of $\Delta \hat{T}$ is limited by the low resistance contrast ( $<10^{2}$ ) of the present VO_2_ film at the metal and insulator phases, and there is room for improvement of the film quality, since previous results have achieved higher resistance contrasts [32, 33].

Although the operating frequency of the metasurface is designed for the available measurement setup, it is straightforward to scale it to lower-frequency regimes such as microwave and millimeter wave. On the other hand, scaling the metasurface to significantly higher frequencies is not trivial, because conductivity of metals decreases as frequency increases and the thickness of the metasurface is not negligible compared to the wavelength. Thus, the theoretical assumption breaks down. However, Babinet’s principle can be extended to plasmonic metasurfaces [43]. It will be an interesting future work to expand the proposed metasurface to higher frequencies.

While the metal–insulator transition of VO_2_ induced by heating is used in this work, there are methods to induce transition electrically [44] and optically [45]. Moreover, other phase-change materials, such as Ge–Sb–Te [46], are interesting alternatives for consideration.

The inversion of the planar-chiral responses can be used to flip the handedness of chiral mirrors dynamically [12, 14], thus reflecting only one handedness state of the circular polarization in a handedness-preserving manner while absorbing the other state on demand. Such chiral mirrors consist of normal mirrors and planar-chiral metasurfaces, so that the proposed dynamic inversion can be used to invert the chiral responses of chiral mirrors at the identical frequencies.
